# Development of a self-report scale to assess relaxation effects of flavors and fragrances

**DOI:** 10.1038/s41598-025-11912-z

**Published:** 2025-07-25

**Authors:** Yoko Hosokawa, Yukio Goto, Kunihide Hoshino, Kensuke Okada

**Affiliations:** 1 Corporate Research & Development Division, Takasago International Corporation, Hiratsuka, Japan; 2 Graduate School of Education, The University of Tokyo, Tokyo, Japan

**Keywords:** Relaxation, Odor, Psychological scale, Nostalgia, Construct validity, Test–retest reliability, Psychology, Human behaviour

## Abstract

Stress is a modern social problem, and it is critical to cope with stress effectively to maintain one’s mental health. Several studies have demonstrated that relaxation helps alleviate depression and anxiety. Although several studies have assessed the relaxation effects of odors, currently, no unified method exists that measures these subjective effects properly and efficiently. To address this gap, we developed the Relaxation Scale of Flavors and Fragrances (RSFF) and examined its structure, reliability, and validity. Two repeated surveys were administered among 110 participants to assess the relaxation effects of flavors and fragrances. The exploratory factor analysis results based on the first survey revealed a three-factor structure as follows: liberation, nostalgia, and negative emotions. Confirmatory factor analysis based on the second survey confirmed this structure. Reliability testing confirmed excellent internal consistency based on Cochran’s alpha, and satisfactory test–retest reliability based on the correlation coefficient between the two repeated surveys’ results. Correlations with the Profile of Mood States revealed satisfactory construct validity. This study’s findings establish the RSFF as a tool for assessing the subjective relaxation effects of flavors and fragrances.

**Supplementary Information:**

The online version contains supplementary material available at 10.1038/s41598-025-11912-z.

## Introduction

Everyday life is stressful. According to Lazarus and Folkman^1^, stress is a mediator between stressors and stress responses, underscoring the importance of recognizing and managing stress. The economy and climate are rapidly changing, and jobs, relationships, and technologies are invariably uncertain. Such environments can cause stress, precipitating mental illnesses such as depression and anxiety disorders and various other health problems. Globally, more than 280 million people suffer from depression and more than 301 million people suffer from anxiety disorders; moreover, mental health problems have worsened in recent years^2^. It has become extremely important for individuals to cope with their daily stress and maintain their mental health. Relaxation is a well-known method for coping with stress. Several studies have confirmed that relaxation alleviates anxiety and depression^3,4^, and arguably, it is extremely important for people to effectively cope with stress and relax to maintain their mental health.

The influence of odors on people’s emotions and moods has been extensively studied, and several studies have demonstrated the relaxation effects of odors. Previous studies have examined issues such as the subjective relaxation effect at specific odor intensities^5^, the implicit relationship between odor and relaxing/energizing^6^, the effect of aromatherapy on anxiety reduction during childbirth^7^, and the effect of odors on improving mood while waiting for dental treatment^8^. Additionally, some reviews exist, including reviews of aromatherapy^9^ and of the relationship between odors and emotions and moods^10^. To efficiently assess the relaxation effects of flavors and fragrances, measuring the subjective relaxation induced by odors is crucial. However, self-report measures that specifically focus on the subjective relaxation effect of flavors and fragrances are lacking.

Currently, the definition of the construct of relaxation varies across studies and is assessed using various measures, such as EEG, heart rate, and subjective assessments^11–15^. Among these, a self-report psychological scale is an attractive method for measuring relaxation effects, as it is inexpensive and relatively easy to use. Several self-report psychological scales have been proposed to measure general relaxation. Examples include the Relaxation Inventory^16^ and Smith Relaxation States Inventory3 (SRSI3)^17,18^. The question items in these scales focus on long-term effects. The Relaxation State Questionnaire by Steghaus and Poth^19^ is an important study in terms of focusing on short-term relaxation. However, as this study focused on the short-term effects of relaxation exercises, it included numerous physical questionnaire items, such as muscle relaxation and breathing, with only two psychological items on the scale. Previous studies regarding the emotions and moods of odors have indicated positive psychological effects^20,21^, and hence, psychological items are expected in a relaxation scale measuring flavors and fragrances to capture the psychological effects. Whether self-report psychological measures of relaxation, which have already been developed, are useful for studying odors is uncertain.

Several self-report scales are available to assess the emotional effects of odors and foods. Numerous studies have employed the State-Trait Anxiety Inventory or Profile of Mood States (POMS) to measure emotions/moods induced by odors^20,22^. However, these scales primarily focus on anxiety and negative moods and may be inadequate to measure the positive psychological effects of odors. Aromatherapy is widely used as a complementary and alternative medicine (CAM) for depression^23^. To evaluate its effectiveness, the Hospital Anxiety and Depression Scale (HADS) is often employed^24,25^. The HADS is a brief 14-item scale that includes some positively worded items. However, because it also contains items related to daily functioning, it may not be sensitive to the short-term relaxation effects induced by olfactory interventions. The Geneva Emotion and Odor Scale (GEOS)^26^ and EsSense Profile®^27,28^ are standard scales used to measure the emotions induced by flavors and fragrances. These exhaustive lists are useful for investigating a wide range of emotions. However, they do not provide sufficient information to assess the degree of subjective relaxation effects in healthy individuals, which vary with the odor. No scale has been developed to comprehensively measure the subjective relaxation effects of odors and their degree of relaxation at present.

As such, this study aimed to develop a new psychological scale to measure the subjective relaxation effects of flavors and fragrances. This study assessed the following three hypotheses through two surveys: First, this scale has a factor structure comprising several correlated factors. Second, this scale has satisfactory test–retest reliability. Third, this scale is related to the POMS subscales. Additional analysis was conducted to verify the internal consistency and analyze the differences in the relaxation effects between the odors.

## Study 1: Development of relaxation scale of flavors and fragrances (RSFF)

### Materials and methods

*Pre-registration* We pre-registered this study with the Open Science Framework (https://osf.io/zpd5y/?view_only=3f0a59470c474ed584b0264cb8e23819). The pre-registered scale’s name was the Relaxation Effect Inventory of Flavors and Fragrances (REIFF), which was then changed to the Relaxation Scale of Flavors and Fragrances (RSFF) to enable wider scale usage.

*Sample* A total of 110 healthy adult Japanese speakers with normal olfactory and cognitive functions were recruited through a research firm. We determined our sample size based on Steghaus and Poth’s study^19^, a previous study that measured relaxation states and recruited 98 participants. Using this sample size as a reference point, we aimed to obtain analyzable data from approximately 100 participants. Given that the home-use-test (HUT) format typically involves a certain dropout level, we recruited 110 participants considering potential attrition. For Study 1, nine participants were excluded owing to noncompliance with the indicated items, resulting in a final sample of 101 participants (50 women and 51 men) with a mean age of 40.73 years (SD = 14.98, age range 18–69 years). Data were collected from participants’ homes using a web-based survey administered by a research firm. Participants who completed both sessions of the repeated survey received a reward of 2,000 Japanese yen. This compensation was explained at the time of recruitment. This study was performed in accordance with the principles of the Declaration of Helsinki on biomedical research involving human subjects. This study’s design was approved by the Ethics Committee of the Takasago International Corporation (Approval number: 202302006R). All the participants provided written informed consent, and data were collected over a one-week period (July 2023).

*Odors* Table 1 presents the odors and labels for each of the five conditions employed in this study. Odors considered to exert a subjective relaxation effect according to previous studies and preliminary research were used. The odor samples were diluted to a subjective equivalent intensity by two people in a preliminary test. Odors, including the no-odor control condition, were presented using lightproof glass bottles. A cotton ball was placed in each glass bottle and 30µL of odor sample was dripped onto it. The lid of each bottle was a screw cap, and the participants opened the lid to sniff the odor during the survey. Each bottle was labeled with a letter of the alphabet (C, P, Q, R, or S)  for each odor sample. Odor samples were sent home before administering the survey, and all participants completed the survey within one week of receiving their odor samples. Odor samples were collected by the research firm after the survey.

**Table 1:** Odors used in the study.

No	Condition	Odors	Label
0	Control	Middle-chain triglycerides (MCT)	C
1	Test	Lemon-lime	P
2	Test	Strawberry	Q
3	Test	Coffee	R
4	Test	Vanilla	S

All odors were provided by Takasago International Corporation. Middle-chain triglycerides are odorless and are often used as solvents in flavors and fragrances.

**Fig. 1:**
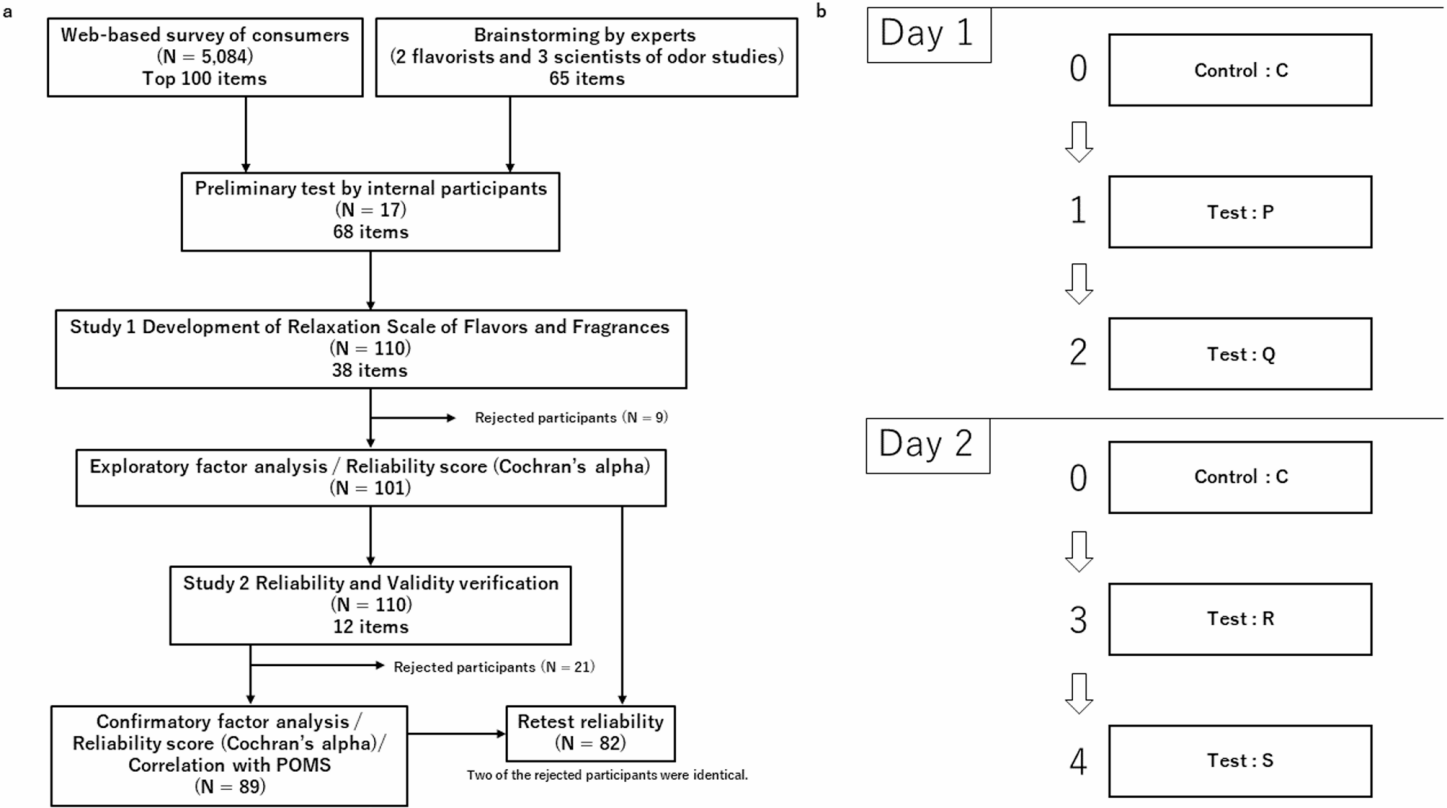
Flowchart and Simplified procedure. (**a**) Presents this study’s flowchart. (**b**) Presents the survey’s protocol. The order of presentation of the four odors in test conditions 1, 2, 3, and 4 was counterbalanced among participants. Accordingly, the order of P, Q, R, and S differed among the respondents.

*Measures* The RSFF prototype. Study 1 aimed to develop and reveal the structure of a scale to measure the subjective relaxation effects induced by odors. As the scale is intended for use in various odor experiments in the future, it mainly aims to assess both the psychological and physical states that change with the presentation of odors. Figure 1a presents the flowchart. First, a set of draft items were collected in two ways. The first was a web-based survey of consumers. We asked consumers to write free-text questions regarding the physical and mental changes that they experienced when they were relaxed by an odor, and conversely, when they were not relaxed by an odor. We collected 5,084 responses. Morphological analysis of the free-text responses was conducted to identify the top 100 words. These words were converted into sentences for use in questionnaires. Another method was expert review, which included two flavorists and three scientists in odor studies from Takasago International Corporation. We assembled odor experts (flavorists) and scientists to incorporate divers perspectives. All experts had over 10 years of professional experience and were selected based on their high motivation and ability to contribute to discussions actively. Although there were only two flavorists, they knew the flavor and fragrance domains, and had professional experience working internationally. The three scientists have specific expertise and experience in psychology and psychophysiology. The scientists involved in the odor studies included two authors (YH and YG). A brainstorming session was held to explain the concepts to be measured in this study and devise the questions to be asked. The two methods were conducted independently, and the experts who participated in the brainstorming were unaware of the consumer results. Further, 65 items were collected through expert review. These were converted into sentences for use as questions. Consequently, many common items were identified between expert brainstorming and consumer survey. Experts generated more items related to physiological changes and emotions, such as familiarity and nostalgia, than consumers.

Subsequently, the following procedures were conducted to organize the collected items: duplicate items were grouped; double-barrel items, items whose meaning was difficult to understand, and general items were removed. This was performed by the author (YH), who organized the collected items from the two methods without any source bias. Consequently, 68 items were retained.

Thereafter, we conducted a preliminary survey of 17 in-house participants using 68 items. The participants were neither flavorists nor perfumers. Participants completed two sessions on separate days. During each session, participants were instructed to smell the odor while completing the 68-item questionnaire, without mentioning relaxation effects. They responded to a 68-item questionnaire three times in a control–test–test sequence. The control condition involved responding without odor, and the test condition involved sniffing the odor while completing the questionnaire. Four different test odors were used, and their order of presentation was randomized across participants. Breaks were introduced as needed to minimize olfactory fatigue during consecutive exposures. The items were rated on a 7-point Likert scale. The 68 items were presented in random order. The analysis sought items that significantly changed the test condition compared with the control condition. Therefore, paired t-tests were performed. Excluding items that changed in an unintended direction, 38 items changed significantly (*p* < 0.05) between the conditions. All items obtained from the preliminary surveys are presented in Appendix A as prototype versions of the RSFF.

In Study 1, along with the 38 items, participants were provided the following instruction: “While sniffing the indicated labeled odor, please rate on a seven-point scale from one (strongly disagree) to seven (strongly agree), how much the statement is accurate to your current feelings and condition right now, at this moment. Please do not think about any statement for too long. Just respond as you feel in the moment.” The order of presentation of the 38 RSFF items was randomized for each participant and odor. To remove dishonest participants, a directed question was presented once a day: “Please respond with 4 to this item.”

Visual Analogue Scale (VAS). The following three items were evaluated using the Visual Analogue Scale (VAS): relaxation, odor liking, and odor intensity—were assessed using a Visual Analogue Scale (VAS). Each item is rated on a 101-point scale. Relaxation was measured from 0 ("not relaxed at all") to 100 (“very relaxed”); odor liking was measured from 0 ("dislike very much") to 100 ("like very much"); and odor intensity was rated from 0 (“very weak”) to 100 (“very strong”). VAS scores were used to validate the RSFF and assess inter-individual variability.

*Design and procedure* Fig. 1b presents the survey procedure. All procedures and instructions in this study were performed in Japanese. The author first translated the questionnaire, then an outside professional proofreading service was asked to make corrections, and the wording was finalized by the author again. The procedure was conducted over two days. On the first day, the participants completed a prototype questionnaire comprising the RSFF and VAS for the control condition. Thereafter, they sequentially sniffed two designated odor samples and completed the same questionnaire for each odor, which comprised the RSFF and VAS. The survey was repeated for each of the odor samples, with the control condition presented at the beginning of the second day, followed by two different odors. The participants were asked to complete a questionnaire that included the RSFF and VAS for each odor. The presentation order of the odors was counterbalanced among the participants. To account for olfactory fatigue caused by the odors, three odor samples—including the control condition—were presented daily. All participants were presented with the control condition at the beginning of the session and responded to questions while sniffing the odor. Subsequently, they responded to the questions in the same manner for the two odors. Participants were randomly assigned to one of 24 possible presentation orders for the odors, excluding the control condition.

*Data analysis* This study deviated from the pre-registration in the following respects: From the standpoint of the results’ interpretability, data from only the test condition were used in the analyses for both Studies 1 and 2, rather than the difference between the control and test conditions. However, data from the control group were used in the analysis of the VAS for manipulation checks. Statistical analyses were performed using R (version 4.1.1) and JASP (version 0.18.1) using the “psych” package for the Kaiser–Meyer–Olkin (KMO) test and eigenvalues, parallel analysis, and Minimum Average Partial (MAP) test. Further, JASP was used for all the other calculations.

## Results

*Exploratory factor analysis (EFA)*　To analyze the data on varied odors simultaneously, the three-dimensional array data of participants, odors, and items were reshaped into two-dimensional data by combining the first two dimensions. This resulted in four different odors per 101 participants, totaling 404 instances of data for each item.

Both the KMO and Bartlett’s tests were run before the EFA. The resultant KMO value exceeded the 0.5 cut-off suggested by Kaiser and Rice^29^, and the Bartlett’s test result was also significant (*p* < 0.001). As these results suggested the data’s appropriateness for factor analysis, exploratory factor analyses were subsequently performed. To determine the number of factors, we used three methods: the scree method, parallel analysis, and MAP. These analyses were performed using R (version 4.1.1). Three, five, and seven factors were supported by the scree method, parallel analysis, and MAP, respectively. We decided on the three factors for interpretability and robustness.

Subsequently, an EFA of all 38 items was performed using JASP (version 0.18.1). As the factors were assumed to be correlated, oblique rotation (promax method) was performed. Owing to the initial analysis, items with high loadings on several factors were removed, based on a threshold factor loading value of 0.35. After repeated factor analysis, a structure comprising three factors was obtained (Appendix B). From the perspective of ease of measurement implementation, 3–5 items were selected from each factor, and a structure comprising 12 items and 3 factors was obtained. Factor loadings ranged from 0.60 to 0.90, and no cross-loading above 0.35 was observed (Table 2).

**Table 2:** Results from the factor analysis of the RSFF.

Item	Factor loading		
	1	2	3
*Factor 1: Liberation*			
7. I feel relieved from stress	**0.90**	−0.02	−0.01
14. I feel lighter in my mind	**0.88**	0.01	0.05
8. My body feels lighter	**0.88**	0.00	0.15
1. I feel comfortable	**0.87**	0.00	−0.11
15. I feel good	**0.84**	0.04	−0.11
*Factor 2: Nostalgia*			
25. I am remembering my childhood	−0.05	**0.86**	−0.02
5. I feel nostalgic	−0.04	**0.86**	−0.09
24. I am remembering my hometown	0.16	**0.72**	0.11
*Factor 3: Negative Emotions*			
22. I am anxious. (R)	−0.07	−0.03	**0.79**
38. I am nervous. (R)	0.12	0.01	**0.70**
21. I am tired. (R)	0.08	−0.06	**0.65**
12. I am bored. (R)	−0.22	0.08	**0.60**

Factor analysis results using oblique rotation (promax method).

Factor loadings of 0.35 or greater are presented in bold. The reversed items are indicated in (R).

Table 3 presents the correlations among the three extracted factors. A positive correlation was observed between Factors 1 and 2, and a negative correlation was observed between Factors 1 and 3.

**Table 3:** Factor correlations for the three factors.

Factor	1	2	3
Liberation	–		
Nostalgia	0.49***	–	
Negative Emotions	−0.38***	0.01	–

Factor correlations derived from EFA. The correlations between Factors 1 and 2 and between Factors 1 and 3 were significant (*p < 0.05). **p < 0.01. ***p < 0.001). All correlations remained significant after the Bonferroni correction.

*Internal consistency reliability* Item scores for the three factors were summed to calculate the subscale scores for liberation (mean = 21.61, SD = 6.67), nostalgia (mean = 10.58, SD = 4.27), and negative emotions (mean = 21.03, SD = 4.15). Cronbach’s alpha was calculated to examine the internal consistency. The scale’s overall reliability was *α* = 0.86. Reliability for the subscales were *α* = 0.94 for liberation, *α* = 0.86 for nostalgia, and *α* = 0.78 for negative emotions. Therefore, the scale’s internal consistency was considered adequate.

## Study 2: Reliability and validity verification

### Materials and methods

*Sample* All 110 participants from Study 1 participated in Study 2. Seven participants were excluded because they did not follow the instructions. This study differed from the pre-registration study in several ways. 14 participants who responded with the same value to all 35 POMS items under identical test conditions were excluded as dishonest respondents. Study 2 had more dishonest respondents than Study 1. This may be attributable to the larger number of questions in Study 2 than in Study 1. Thus, Study 2’s final sample comprised 89 participants (44 women and 45 men, mean age 41.56 years, SD = 15.02, age range 18–69 years). Additionally, the test–retest reliability validation sample comprised 82 participants (41 women and 41 men; mean age 40.77 years, SD = 15.11, age range 18–69 years) who were honest respondents in both Studies 1 and 2.

Data for Study 2 were collected over a one-week period (August–September 2023)—seven weeks after Study 1. Many previous studies have assessed test–retest reliability with intervals ranging from approximately 1 to 11 weeks^30^. In this study, a 7-week interval was selected as a suitable timeframe for the repeated participation of participants.

*Odors* We used the same odors in Study 2 as those in Study 1. Moreover, the odor presentation method was the same. Freshly prepared odor samples were sent home again before administering the survey, and all participants completed the survey within two weeks of adding the odors to their cotton balls. Odor samples were collected at the survey period’s end.

*Measures* Relaxation Scale of Flavors and Fragrances (RSFF). All 12 items from Study 1 were used and are listed in Table 2. The response method was the same as that in Study 1.

Visual Analogue Scale (VAS). The items and response methods were the same as those in Study 1.

Profile of Mood States (POMS). The *Japanese Translation of POMS®2: Profile of Mood States Second Edition* for adults is a 35-item questionnaire that assesses mood on a 5-point Likert scale^31^. The POMS score is based on the Anger–Hostility (AH), Confusion–Bewilderment (CB), Depression–Dejection (DD), Fatigue–Inertia (FI), Tension–Anxiety (TA), and Vigor–Activity (VA) subscales, and the Total Mood Disturbance (TMD) score was calculated using the following formula: [(AH) + (CB) + (DD) + (FI) + (TA)-(VA)].

*Design and procedure* As Fig. 1b indicates, the same procedure was used as that in Study 1, but the participants responded to the RSFF and VAS, followed by the POMS2 short version.

*Data analysis* As in Study 1, statistical analyses were performed using R (version 4.1.1) and JASP (version 0.18.1).

### Results

*Confirmatory factor analysis*　To analyze the data for varied odors simultaneously, the three-dimensional array data of participants, odors, and survey results were reshaped into two-dimensional data by combining the first two dimensions. This resulted in four different odors for each of the 89 participants, totaling 356 instances of data for each item.

**Fig. 2:**
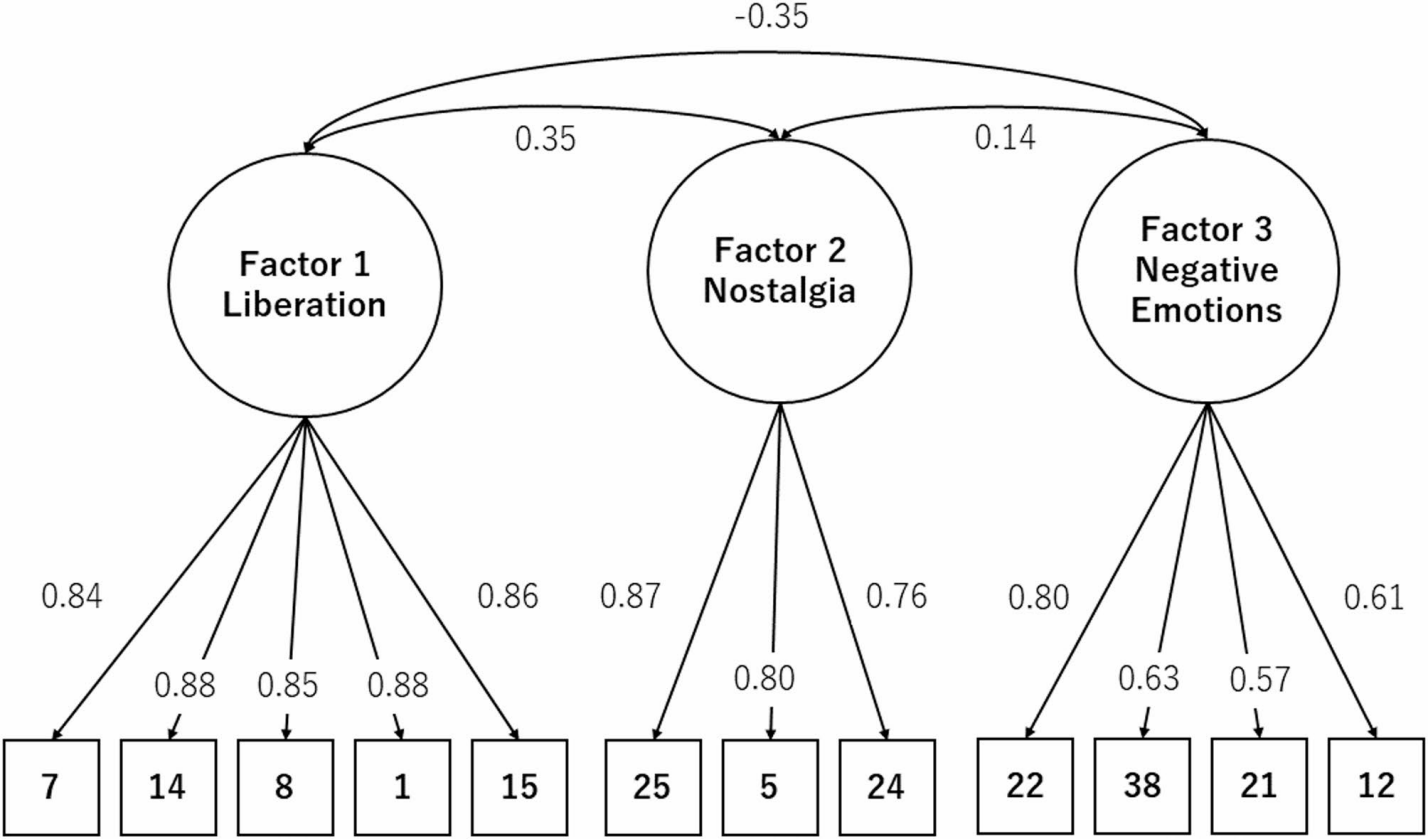
Path diagram of the confirmatory factor analysis model with parameter estimates The question item numbers correspond to those listed in Table 2. All parameter estimates are standardized.

Both the KMO test and Bartlett’s test were performed before the EFA. The resultant KMO value exceeded the 0.5 cut-off suggested by Kaiser and Rice, and the Bartlett’s test result was also significant (*p* < 0.001). Therefore, using the factor structure identified in the EFA, a confirmatory factor analysis was conducted on the 12 items. Figure 2 presents the path diagram and resultant factor loading values of the RSFF, as well as the correlations between the three scales. The result indicated that the factor loadings of the items ranged from 0.57 to 0.87.

The goodness-of-fit indices for the confirmatory factor analysis were Comparative Fit Index (CFI) = 0.95, Tucker-Lewis Index (TLI) = 0.93, and Root Mean Square Error of Approximation (RMSEA) = 0.08, which are relatively satisfactory values. That is, the three-factor structure hypothesized in this study was supported by the data.

*Internal consistency reliability* Cronbach’s alpha was calculated to examine internal consistency. This scale’s overall reliability was *α* = 0.82. The subscales’ reliability were α = 0.93 for liberation, *α* = 0.85 for nostalgia, and *α* = 0.75 for negative emotions. Therefore, the scale’s internal consistency was adequate.

**Fig. 3:**
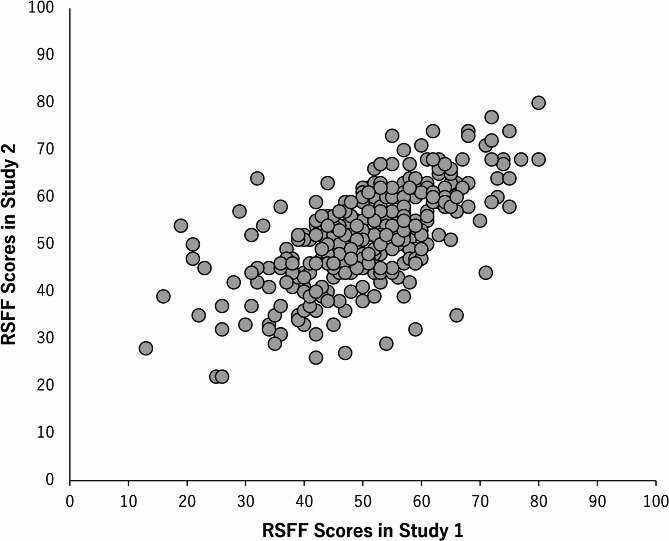
Test–retest reliability. The horizontal axis presents the RSFF scores of Study 1, while the vertical axis presents the RSFF scores of Study 2. The correlation coefficient between the two scores was *r* = 0.65, which was statistically significant (*p* < 0.001).

*Test–retest reliability* To examine test–retest reliability, we used data from 82 participants who were common to Studies 1 and 2 for this analysis. We used 328 instances of data for four different odors from 82 participants. Figure 3 presents the RSFF scores for the two surveys. Pearson’s correlation coefficient (*r*) was calculated for the RSFF scores between Studies 1 and 2. The resultant correlation coefficient was *r* = 0.65, which was moderately strong and statistically significant (*p* < 0.001). Therefore, the test–retest reliability was confirmed.

*Correlations with POMS* To examine the RSFF’s validity, correlation coefficients with the POMS were examined. Table 4 presents the correlations between the RSFF and subscales of the POMS short version. A floor effect was observed on the negative subscale of the POMS. Therefore, the correlation coefficient between VA and other negative subscales identified in a previous study^29^ was not significant. This may be because the current study only sniffed odors without the negative emotion elicitation task. Further, RSFF scores and F1 (liberation) were positively correlated with VA scores, and F3 (negative emotions) was correlated with all POMS subscales. Moreover, F2 (nostalgia) did not correlate with any of the POMS subscale scores. This result was unexpected; however, as nostalgia has also been described as a bittersweet emotion^32^, it may be a complex emotion with a combination of positive and negative emotions that cannot be captured by the POMS.

**Table 4:** Correlation with POMS.

	RSFF					POMS						
	RSFF	F1 Liberation	F2 Nostalgia	F3 Negative Emotions		AH Anger–Hostility	CB Confusion–Bewilderment	DD Depression–Dejection	FI Fatigue–Inertia	TA Tension–Anxiety	VA Vigor–Activity	TMD
RSFF	–											
F1	0.87***	–										
F2	0.58***	0.32***	–									
F3	−0.51***	−0.25***	0.12	–								
AH	0.02	0.10	0.12	0.23***		–						
CB	−0.09	0.02	0.09	0.37***		0.81***	–					
DD	−0.02	0.08	0.14	0.33***		0.71***	0.87***	–				
FI	−0.08	0.04	0.07	0.33***		0.77***	0.88***	0.78***	–			
TA	−0.07	0.06	0.10	0.38***		0.79***	0.91***	0.84***	0.77***	–		
VA	0.35***	0.32***	−0.02	−0.29***		−0.02	−0.10	−0.12	−0.11	−0.05	–	
TMD	−0.14	−0.03	0.11	0.41***		0.83***	0.94***	0.89***	0.91***	0.92***	−0.32***	-

All correlations were tested for uncorrelation (*p < 0.05, **p < 0.01, ***p < 0.001).

All of the *p*-values were calculated using Bonferroni correction.

**Fig. 4:**
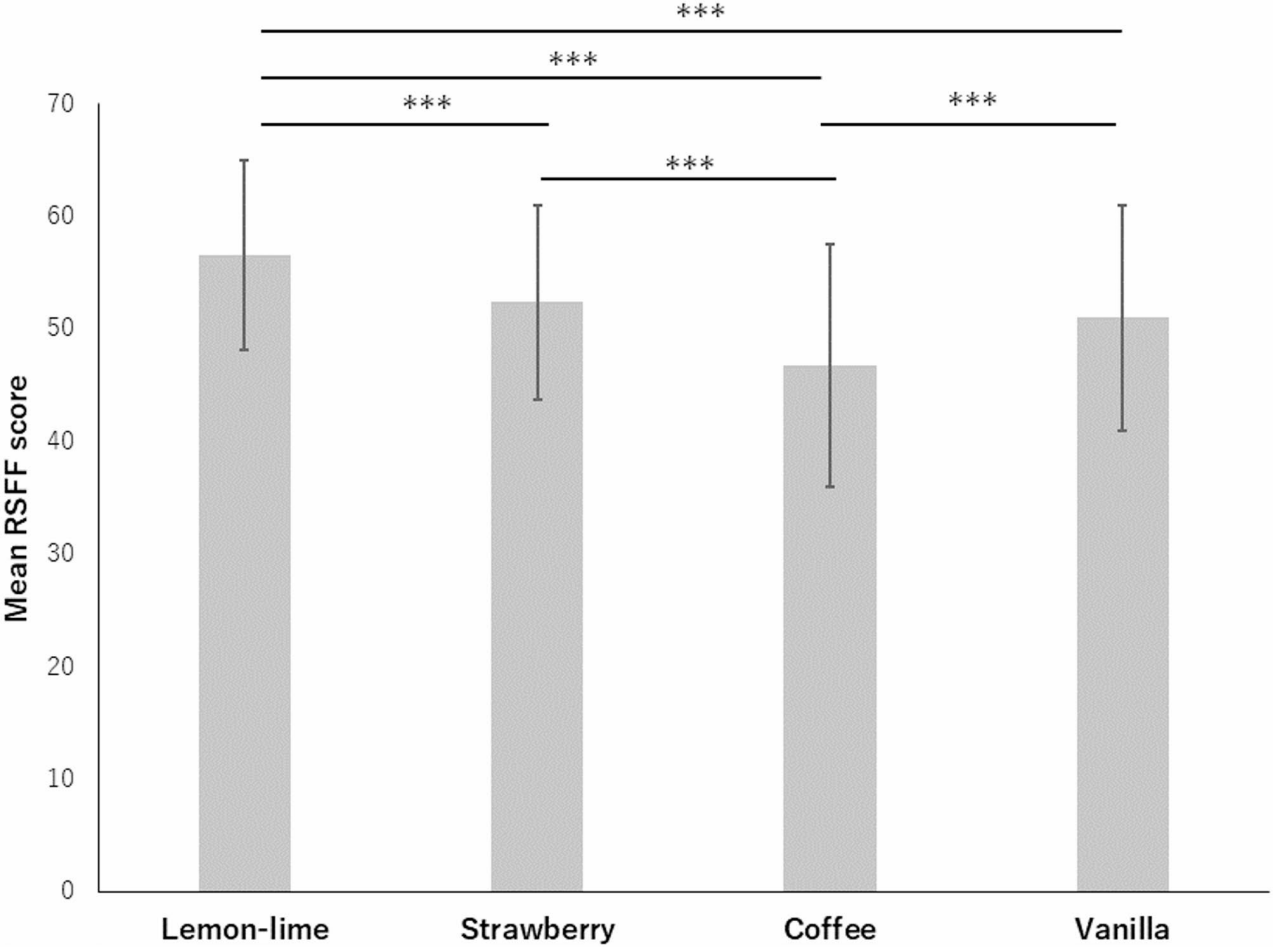
Mean and standard deviation of RSFF scores. Results of multiple comparisons (*p < .05. **p < .01. ***p < .001.) All *p*-values were calculated using Bonferroni correction.

*Relaxation Degree Analysis* Additional analysis was conducted to examine whether the RSFF successfully captured the degree of relaxation. Figure 4 presents the mean and standard deviation in the RSFF score for each odor. Multiple comparisons of the RSFF scores were performed to determine whether there were differences in the subjective relaxation effects of the four relaxing odors. All p-values were Bonferroni-corrected. The score was significantly higher for lemon-lime odors than for the other three odors. Similar results were obtained when relaxation was measured using VAS (Appendix C). Additionally, a positive correlation was observed between RSFF and VAS scores (*r* = 0.73). The coefficient of variation (CV) values, which are the standard deviation divided by the arithmetic mean and indicate the degree of variability in the data, were calculated for both RSFF and VAS scores (Appendix D). The resultant CV values of the RSFF score were consistently lower than those of the VAS score for all odors, indicating that by measuring the construct of relaxation using a psychometric scale, the RSFF enables less variable and more consistent measurements than the VAS scale. This may reflect individual differences in the interpretation of a single VAS scale. These results suggest that the RSFF scale developed in this study effectively reflects the subjective degree of relaxation induced by odors.

## Discussion

This study aimed to develop a psychological scale to measure the relaxation effects of flavors and fragrances. To this end, we determined its structure and tested its reliability and validity. In Study 1, EFA was conducted to determine the scale’s structure, which comprised three factors. In Study 2, the three-factor model was validated by conducting a confirmatory factor analysis. We confirmed high factor loading values, a satisfactory model fit, and high internal consistency reliability. Test–retest reliability was confirmed using two repeated surveys. Correlations with the subscales of the POMS—a well-established scale—were also examined. The analysis results indicated that the scale measuring the relaxation effect induced by odors is a valid instrument with high reliability and validity. Moreover, this scale was found to provide a stable measurement of the degree of subjective relaxation.

The RSFF’s factor structure was examined and a structure comprising three factors was identified: liberation, nostalgia, and negative emotions. Additionally, these factors were correlated with each other. Items similar to those in factors 1 and 3 were also observed on a previously reported relaxation scale^16–18^. Factor 1, liberation, comprises relief from stress, lightness of the body and mind, and positive emotions; the etymology of the word “relax” comes from the Latin word “relaxare”, that is, “re”(again) and “laxare”(to loosen). Factor 1 reflected the essential meaning of relaxation. The essential meaning of relaxation can be measured using several items, including freedom from stress, reduction of stiffness in the body and mind, and positive emotions. Factor 3, negative emotions, reflects anxiety and tension, which are antonyms for relaxation. The negative emotions resulting from stress can be measured using several items. Noteworthily, the nostalgia factor in Factor 2 was specific to the RSFF; a question item in the SRSI3 has similar items, such as “I feel innocent and childlike,” but no clear nostalgia factor was observed in the other relaxation measures. On the contrary, the GEOS and EsSense® profiles, which are designed to assess subjective emotions specific to odors and foods, use a questionnaire item called “nostalgic.” Nostalgia may be an inseparable emotion when studying emotions induced by odors and foods. A well-known phenomenon associated with odors and memory is the Proust phenomenon of evoking past memories and emotions^33^. Odors are believed to have the power to induce vivid mental time travel. It may be credible that nostalgia is partially related to the subjective relaxation effects of flavors and fragrances, as it has been suggested that odor-induced nostalgia may be related to positive emotions and self-esteem^34^. Moreover, if relaxation is the opposite of the fight-flight response to stress^35,36^, the idea that familiar nostalgic odors that we have smelled in the past are likely to be safe and, therefore, related to the relaxation response may be accurate. However, the factor observed in the scale developed herein was personal nostalgia as inferred from the questionnaire items. Therefore, it can be inferred that this varies according to the individual experience and history. Nostalgia may influence individual differences in RSFF scores. However, this factor’s inclusion strongly reflected the scale’s specificity in the odor context.

This scale’s reliability was satisfactory. The values of its alpha coefficients were all high, confirming its internal consistency. Furthermore, test–retest reliability was tested by administering two tests seven weeks apart, and a moderately strong and statistically strong correlation was found between the two repeated surveys, confirming test–retest reliability. The correlation coefficient value was not extremely large (0.65), which is attributable to the fact that the RSFF assumes a short-term “state” rather than a long-term trait. The RSFF’s validity was tested by correlating it with an existing mood scale. Overall, the RSFF was associated with a positive subscale on the POMS VA. Therefore, this scale may strongly indicate the positive effect of relaxation.

This study has certain limitations regarding the generalizability of the findings. The sample included men and women various age groups, and all participants were native Japanese speakers living in Japan. The subjective experience involves substantial individual variation and is strongly influenced by cultural background^37^. Therefore, caution should be exercised when generalizing these results to broader or cross-cultural populations. Despite this limitation, this study has a strong ecological validity, because it involved participants who physically experienced odors during the assessment process. This design enhances the relevance of the findings to the intended population for which a psychological scale is being developed. Future research should aim to validate and extend the present findings using larger and more diverse samples, including participants from different cultural backgrounds, to evaluate the scale’s generalizability and applicability further.

There may be some questions about whether our sample size was sufficient for exploratory factor analysis. In the literature on EFA, the absolute number of sample size (Ns) ranges from 100 to 250^38^, 300^39^, and over 500^40^. In our study, the number of participants was 101, and the repetition of the four odor conditions yielded 404 data for analysis. This is sufficient considering the results of previous studies. Another category of empirical rules is ratios, in which N:p ratios are used, participants (N) and items (p) are traditionally 5:1^40,41^. The N:p ratio in the EFA in this study was 404 (N):38 (p), or approximately 11:1. This was considered appropriate based on previous studies. However, as the data contained individual repetitions, creating a model that accounts for individuals in the future may be necessary. The reliability of the RSFF was also confirmed by the fact that the factor loadings for all items ranged from 0.6 to 0.9 and confirmatory factor analysis reproduced the initial findings. Collectively, these results are considered robust. In this study, we did not assess the relationship between several previously proposed self-report psychometric scales used to measure general relaxation and a standard list of emotional words adapted to odors and foods. Additionally, numerous psychophysiological methods have been proposed to measure relaxation^10–13^. Whether the developed scale is related to the results of measuring the relaxation effects of odors using these methods has not been tested. Although the relationships between the developed measures and the VAS and POMS were examined in this study, further investigating how the present scale correlates with other psychological scales and psychophysiological methods would be beneficial. Because the RSFF is a brief 12-item scale, it can be easily administered between physiological assessments such as EEG or heart rate monitoring, which are commonly used as objective indicators of relaxation. Therefore, conducting these measurements simultaneously may help explore the relationship between relaxation’s subjective and physiological aspects. Moreover, while this study measured the subjective relaxation effect of orthonasal aroma, a subsequent research question is whether it can be applied to the relaxation effect experienced when consuming food and drinks (related to retronasal aroma). A preliminary web survey of 1,045 respondents asked them to report when they experienced subjective relaxation while consuming food and beverages (Appendix E). The results indicated that approximately 58% of respondents answered when they were sniffing the odor of the food or beverage before eating it. Therefore, exploring whether similar results can be obtained under retronasal conditions would be beneficial. Finally, as this scale focuses on short-term effects, re-examining how short-term relaxation effects are related to long-term mood is also possible. Emotions and moods differ in persistence and intensity^42^. The present measurement covered short-term emotions. Conversely, mood disorders, such as depression, are related to long-term mood. It is important to consider how the temporary relaxation effects of the odors measured in this study affect long-term mood and well-being. By combining the HADS with our scale specifically designed to measure short-term relaxation responses to odors, it may be possible to elucidate how short-term effects of aromatherapy interventions influence long-term anxiety and depressive symptoms.

We developed a psychological scale to assess the subjective relaxation effects of flavors and fragrances. This scale’s structure is clear, with satisfactory factor loadings and reliability. Furthermore, this scale’s validity was confirmed through correlations with established methods. The questionnaire comprises a short list of 12 items with a stable structure. Moreover, it is a sensitive and stable measure of subjective relaxation. Subjective ratings can be easily administered in various research settings and are likely to contribute to research on the relaxation effects of flavors and fragrances. For example, the RSFF can be administered alongside physiological measures, such as EEG and heart rate monitoring in research settings. In the clinical context, it allows the evaluation of short-term effects of aromatherapy interventions. Beyond clinical use, the RSFF also holds the potential for use in everyday life as a tool for individuals to assess the effectiveness of their own self-care practices. This may contribute to advancing research in this field and benefit individuals’ well-being. Odors can be applied to varied products and can be an easy stress-management tool that can be used anytime and anywhere. By helping people use odors effectively, we can contribute to a future wherein they can manage their emotions appropriately, thereby enhancing their well-being.

## Electronic supplementary material

Below is the link to the electronic supplementary material.

Supplementary Material 1

## Data Availability

The datasets analyzed during the current study are not publicly available because of the privacy of the participants but are available from the corresponding author upon reasonable request.
