# Supplementary material for: Development of a self-report scale to assess relaxation effects of flavors and fragrances
Source: Sci Rep. 2025 Jul 25;15:27110. doi: 10.1038/s41598-025-11912-z (PMC12297476; doi:10.1038/s41598-025-11912-z)
Supplement: Supplementary file 1 — Supplementary Material 1 [file 41598_2025_11912_MOESM1_ESM.docx]

**Supplementary Information**

| Item | Japanese | English |
| --- | --- | --- |
| 1 | 心地よい | I feel comfortable |
| 2 | 安心している | I feel at ease |
| 3 | 集中している | I am concentrated |
| 4 | 満たされた気持ちだ | I feel satisfied |
| 5 | 懐かしい気持ちだ | I feel nostalgic |
| 6 | 今の状態のままでいたい | I want to stay present |
| 7 | ストレスから解放された感じがする | I feel relieved from stress |
| 8 | 体が軽くなった感じがする | My body feels lighter |
| 9 | 体の力が抜けている | My body feels more relaxed |
| 10 | いつもより深く呼吸している | I am breathing deeper than usual |
| 11 | いつもより体温が上がった感じがする | I feel my body temperature is higher than usual |
| 12 | 退屈だ | I am bored |
| 13 | 肩の力が抜けている | My shoulders feel relaxed |
| 14 | 心が軽くなった感じがする | I feel lighter in my mind |
| 15 | 気分が良い | I feel good |
| 16 | 心身が癒されている | My body and mind are healed |
| 17 | やる気がある | I feel motivated |
| 18 | 頭がすっきりしている | My head is clear |
| 19 | 優しい気持ちだ | I feel gentle |
| 20 | 気分が上がる | I feel uplifted |
| 21 | 疲れている | I am tired |
| 22 | 不安だ | I am anxious |
| 23 | 馴染みのある感じがする | I feel familiarity |
| 24 | ふるさとを思い出すような感じがする | I am remembering my hometown |
| 25 | 小さい頃を思い出すような感じがする | I am remembering my childhood |
| 26 | 良い経験を思い出すような感じがする | I am remembering a good experience |
| 27 | 爽やかな感じがする | I feel refreshed |
| 28 | 新鮮な感じがする | I feel freshness |
| 29 | 豊かな感じがする | I feel richness |
| 30 | あたたかい感じがする | I feel warmth |
| 31 | 調和のとれた感じがする | I feel harmony |
| 32 | 明るい感じがする | I feel brightness |
| 33 | 甘い感じがする | I feel sweetness |
| 34 | 複雑な感じがする | I feel complexity |
| 35 | 濃厚な感じがする | I feel fullness |
| 36 | 澄んだ感じがする | I feel clearness |
| 37 | 奥行のある感じがする | I feel depth |
| 38 | 緊張している | I am nervous |

**Appendix A.** List of Japanese question items and English translation.

List of 38 question items from the RSFF prototype used in Study 1 in Japanese and translated into English.

|  | Item | Factor Loading | | |
| --- | --- | --- | --- | --- |
|  |  | 1 | 2 | 3 |
| Factor 1: Liberation | | | | |
| ✓ | 7． I feel relieved from stress | **0.92** | -0.08 | 0.00 |
| ✓ | 8． My body feels lighter | **0.91** | -0.06 | 0.16 |
| ✓ | 1． I feel comfortable | **0.89** | -0.05 | -0.11 |
| ✓ | 14． I feel lighter in my mind | **0.89** | -0.04 | 0.04 |
| ✓ | 15． I feel good | **0.88** | -0.02 | -0.10 |
|  | 4． I feel satisfied | **0.88** | 0.03 | -0.10 |
|  | 6． I want to stay present | **0.86** | -0.09 | 0.04 |
|  | 20． I feel uplifted | **0.84** | 0.04 | 0.09 |
|  | 16． My body and mind are healed | **0.83** | 0.02 | -0.04 |
|  | 2． I feel at ease | **0.82** | 0.08 | -0.11 |
|  | 10． I am breathing deeper than usual | **0.81** | -0.08 | -0.05 |
|  | 32． I feel brightness | **0.78** | 0.00 | -0.02 |
|  | 31． I feel harmony | **0.76** | 0.12 | 0.00 |
|  | 17． I feel motivated | **0.73** | 0.03 | -0.04 |
|  | 29． I feel richness | **0.71** | 0.10 | 0.12 |
|  | 19． I feel gentle | **0.69** | 0.16 | 0.02 |
| Factor 2: Nostalgia | | | | |
| ✓ | 5． I feel nostalgic | -0.04 | **0.86** | -0.09 |
| ✓ | 25. I am remembering my childhood | -0.04 | **0.85** | 0.00 |
| ✓ | 24． I am remembering my hometown | 0.16 | **0.71** | 0.12 |
| Factor 3: Negative Emotions | | | | |
| ✓ | 22． I am anxious (R) | -0.09 | -0.01 | **0.78** |
| ✓ | 38． I am nervous (R) | 0.13 | 0.01 | **0.71** |
| ✓ | 21． I am tired (R) | 0.09 | -0.06 | **0.65** |
| ✓ | 12． I am bored (R) | -0.23 | 0.10 | **0.60** |

**Appendix B.** Results from the factor analysis of the RSFF of 23 items.

Results of exploratory factor analysis. Considering the ease of implementation, 3-5 items with ✓ were selected from each factor and exploratory factor analysis was conducted in the text.


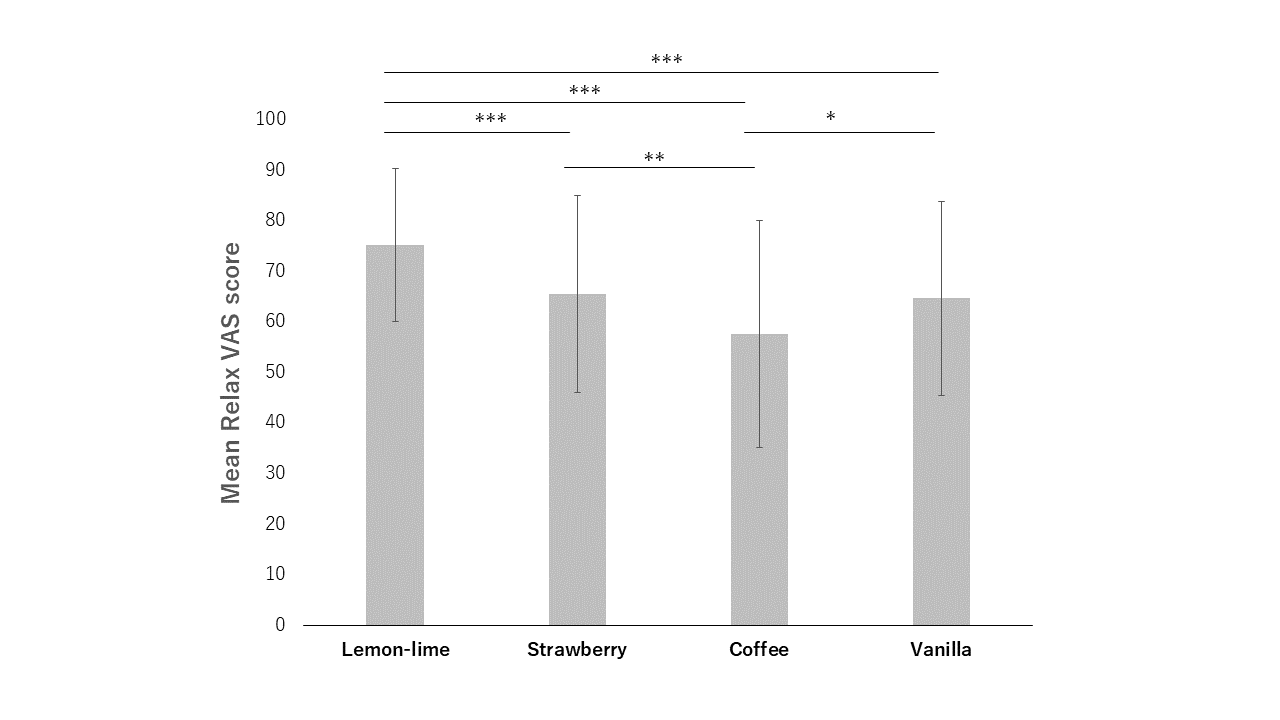


**Appendix C.** Mean and standard deviation of VAS scores.

Results of multiple comparisons (**p* < .05. ***p* < .01. ****p*< .001.).

All *p*-values were calculated using Bonferroni correction.

|  | Lemon-lime | Strawberry | Coffee | Vanilla |
| --- | --- | --- | --- | --- |
| RSFF score | 0.15 | 0.16 | 0.23 | 0.20 |
| Relax VAS score | 0.20 | 0.30 | 0.39 | 0.30 |

**Appendix D.** Coefficient of variation of RSFF scores and VAS scores

CV value is the standard deviation divided by the arithmetic mean and is a measure of relative variability. Compared to the VAS, the RSFF score consistently shows smaller CV values. This suggests that RSFF has less variability and enables more consistent measurement.

**Appendix E.** Preliminary web survey results.

Preliminary web survey of 1,045 Japanese speakers. The respondents were asked to choose one of three options for when to subjective relaxation by eating food and drink. The results showed that about 58% of respondents answered while they are sniffing the odor of the food or beverage before eating it.
